# Remote Foot Temperature Monitoring Among Veterans: Large Observational Study of Noncompliance and Its Correlates

**DOI:** 10.2196/53083

**Published:** 2024-11-05

**Authors:** Alyson J Littman, Andrew K Timmons, Anna Korpak, Kwun C G Chan, Kenneth T Jones, Suzanne Shirley, Kyle Nordrum, Jeffrey Robbins, Suhail Masadeh, Ernest Moy

**Affiliations:** 1 Seattle ERIC, VA Puget Sound Health Care System, 1660 S Columbian Way, S-152E, Seattle, WA, 98108, United States, 1 206-277-4182; 2 Seattle-Denver Center of Innovation for Veteran-Centered and Value-Driven Care, VA Puget Sound Health Care System, Seattle, WA, United States; 3 Department of Epidemiology, University of Washington, Seattle, WA, United States; 4 Department of Biostatistics, University of Washington, Seattle, WA, United States; 5 VA Office of Health Equity, Department of Veterans Affairs, Washington, DC, United States; 6 VHA Innovation Ecosystem, Department of Veterans Affairs, Washington, DC, United States; 7 Podiatry Program Office, Department of Veterans Affairs Central Office, Specialty Care Services, Washington, DC, United States; 8 Department of Surgery, Division of Podiatry, Cincinnati Veteran Affairs Medical Center, Cincinnati, OH, United States

**Keywords:** diabetes, self-monitoring, ulceration, compliance, foot temperature monitoring, SmartMat, adherence, remote, monitoring, non-compliance, foot, ulcer, diabetic, veteran, observational, health record, EHR, noncompliance, electronic health record

## Abstract

**Background:**

In-home remote foot temperature monitoring (RTM) holds promise as a method to reduce foot ulceration in high-risk patients with diabetes. Few studies have evaluated adherence to this method or evaluated the factors associated with noncompliance.

**Objective:**

The aims of this study were to estimate noncompliance in patients who were enrolled in RTM nationwide across Department of Veterans Affairs (VA) and to evaluate characteristics associated with noncompliance.

**Methods:**

We conducted an observational study including 1137 patients in the VA who were enrolled in RTM between January 2019 and June 2021, with follow-up through October 2021. Patient information was obtained from the VA’s electronic health record and RTM use was obtained from the company. Noncompliance was defined as using the mat <2 days per week for ≥4 of the 12 months of follow-up. Using a multivariable model, we calculated odds ratios (ORs) and 95% CIs for associations between various factors and noncompliance and compared using Akaike information criterion statistics, a measure of model fit.

**Results:**

The sample was predominantly male (n=1125, 98.94%) ; 21.1% (n=230) were Black and 75.7% (n=825) were White. Overall, 37.6% (428/1137) of patients were classified as noncompliant. In the multivariable model, an intermediate area deprivation index was statistically significantly and inversely associated with noncompliance (area deprivation index 50‐74 vs 1‐24; OR 0.56, 95% CI 0.35-0.89); factors significantly and positively associated with noncompliance included recent history of osteomyelitis (OR 1.44, 95% CI 1.06-1.97), Gagne comorbidity index score ≥4 (vs ≤0; OR 1.81, 95% CI 1.15-2.83), telehealth encounters (28+ vs <6; OR 1.70, 95% CI 1.02-2.84), hemoglobin A_1c_≥10 (vs <5.7; OR 2.67, 95% CI 1.27-5.58), and current smoking (OR 2.06, 95% CI 1.32-3.20). Based on Akaike information criterion differences, the strongest factors associated with noncompliance were behavioral factors (poor glucose control [as measured by hemoglobin A_1c_] and smoking), and to a lesser extent, factors such as a recent history of osteomyelitis and an elevated Gagne comorbidity index, indicating a high comorbidity burden.

**Conclusions:**

To reduce the risk of ulcer recurrence and amputation, proactively providing additional support for self-monitoring to patients with characteristics identified in this study (poor glucose control, current smoking, high comorbidity burden) may be helpful. Furthermore, research is needed to better understand barriers to use, and whether the addition of design features, reminders, or incentives may reduce noncompliance and the risk of foot ulcers.

## Introduction

Diabetic foot ulcers are common, debilitating, and costly diabetes complications. Over 10 percent of US adults [1] and nearly a quarter of veterans enrolled in Veterans Health Administration (VHA) have diabetes [2]. In patients with diabetes, lifetime risk of ulceration is estimated to be 14% [3]. Ulcerations negatively impact mobility, mental health, and quality of life, and have high recurrence rates. Nearly two-thirds of patients have a recurrence within 5 years of ulcer healing [4]. Loss of pain sensitivity, foot deformity, and peripheral artery disease place individuals at high initial and subsequent risk of ulceration; these conditions do not resolve after healing.

Five systematic reviews and meta-analyses [5-9] have been conducted that each included the same 4 or 5 [10-14] randomized controlled trials of foot temperature monitoring. While there were slightly different analytic approaches in each meta-analysis, all estimated a substantially lower risk of ulceration in the groups assigned to monitor plantar foot temperatures compared to the usual care groups (meta-analysis odds ratios [ORs] or relative risks ranged from 0.37 [7] to 0.53 [8]). Based on this research, several clinical organizations have endorsed foot temperature monitoring [15-17], but it is rarely practiced because measuring temperatures on multiple parts of feet daily and then calculating differences between the feet is time consuming and burdensome. New technologies, including temperature sensing mats, have eliminated much of the burden, and made foot temperature monitoring easier [18,19].

In 2019, the VHA, the largest integrated health care system in the United States, began national implementation of remote foot temperature monitoring (RTM) using SmartMats. The VHA Innovation Ecosystem launched the Initiative to End Diabetic Limb Loss [20] in partnership with the VHA Podiatry Service, Office of Health Equity and Office of Connected Care to design new care models that incorporated emerging technologies such as the SmartMat in early detection of diabetic foot ulcers.

We are aware of a single study that published data on compliance with use of the SmartMat [21]. This study included 132 people with diabetes and a prior diabetic foot ulcer who were recruited from 7 outpatient sites in the United States. During 34 weeks of follow-up, patients used the device 5.0 days per week on average (SD not reported) and 86% of patients used the SmartMat ≥3 times per week [21]. Data on characteristics associated with use were not reported.

Understanding compliance outside of a study is valuable to assess the real-world potential for effectiveness. Additionally, identifying which patients might be less likely to comply with RTM recommendations could be helpful to identify barriers and determine which patients might benefit from additional support to improve compliance. Thus, the aims of this study were to estimate noncompliance in patients who were enrolled in RTM nationwide across VHA between 2019 and 2021 and to evaluate characteristics associated with noncompliance.

## Methods

### Study Data

Demographic, geographic, medical, and use data came from the corporate data warehouse (CDW). Race and ethnicity are determined based on self report. During this study’s period, when a patient was enrolled in RTM, their Department of Veterans Affairs (VA) provider placed an order for the device through the Prosthetics Department. As the company making the devices only had 1 product, we were able to determine enrollment in RTM based on the Data Universal Numbering System number (DUN & Bradstreet). The company provided information on the average number of times per week that patients used the mat each calendar month from January 2019 until October 2021.

### Study Population

We included individuals who were enrolled in RTM in VA between January 2019 and June 2021 (as identified in the CDW) and for whom we were also able to obtain SmartMat use data. Of those enrolled in January 2019 or later (n=1675), we were able to link 1641 individuals to other data in the CDW. We excluded 504 people with less than 12 months of follow-up, leaving 1137. Due to how the variables were constructed, individuals for whom we could not determine a home facility (n=123) were included in most analyses, but were missing for area deprivation index (ADI), VA district or region, and facility complexity.

### RTM in VA

The device under investigation is a daily-use telemedicine foot temperature monitoring SmartMat made by Podimetrics. Patients with a high risk of ulceration (mainly due to a history of foot ulceration; lower limb, foot, or toe amputation; or Charcot foot) are eligible for RTM (Kyle Nordrum, DPT, personal communication). The device is ready to use without any configuration or set-up. A temperature scan takes 20 seconds, and the temperature data are transmitted to the cloud using an embedded cellular component. All scans are timestamped, allowing for objective measures of use. The software detects “hot spots,” defined as asymmetries of ≥2.2˚C between the same region on the left and right foot or different regions on the same foot. Temperature asymmetries that persist for at least 2 days are predictive of ulceration [21]. When hot spots are detected, either clinical support staff from the company or the VA provider follows-up with the patient to evaluate risk factors and make recommendations about actions to take. Patients are also called if they fail to use the mat for several days in a row to assess reasons for nonuse and encourage re-engagement.

### Definition of Noncompliance

While daily use is recommended, using the device at least 2 times per week is thought to be sufficient for the detection of hot spots (Jon Bloom, MD, personal communication). As we had a relatively long follow-up (12 mo), we defined noncompliance as using the mat less than 2 times per week for at least a quarter of the months under observation. Specifically, patients were considered noncompliant if they used the mat less than 2 days per week for 4 or more months out of 12.

### Compliance Correlates or Predictors

We evaluated demographic, geographic, clinical, and facility factors, as well as health care use as potential correlates of nonadherence. Details about the data sources, definitions, and categories are included in Table 1.

**Table 1.** Data definitions, categories, and sources.

Domain, variable	Categories	Data sources, timing, and other details
**Demographics**		
Sex	Male or female	CDW^a^; assumed to be sex assigned at birth
Race	American Indian or Alaska Native, Asian, Black or African American, Native Hawaiian or Pacific Islander, White, or more than 1 race	CDW, based on self report
Hispanic or Latinx ethnicity	Yes or no	CDW, based on self report
**Geographic access**		CDW; drive distance and drive time used the most recent data (FY14-FY19^b^)
Drive time to primary care	<30 minutes or ≥30 minutes	Estimated drive time between the coordinates of the primary care facility nearest to the veteran’s home and the veteran’s home address.
Drive time to specialty care	<60 minutes or ≥60 minutes	Estimated drive time (network distance) between the coordinates of the specialty care facility nearest to the veteran’s home and the veteran’s home address.
Rurality	Urban, rural, or highly rural	Determined using RUCA^c^ codes, which are based on zip code approximations. RUCA codes of 10=highly rural; RUCA codes of 1 or 1.1=urban. All other codes were considered rural [22].
VA^d^ districts	Continental, Midwest, North Atlantic, Pacific, and Southeast	
**Area level factors**		
Area Deprivation Index (ADI)	Quartiles—lower ADI^e^ indicates *less* deprivation	The ADI is a measure of socioeconomic resources and well-being that includes factors for income, education, employment, and housing quality. The ADI has been adapted and validated to the Census Block Group [23] and allows for rankings of neighborhoods by socioeconomic disadvantage at the national level.
**Clinical characteristics**		CDW; ascertained in the 2 years prior to baseline
Other conditions: Chronic kidney disease or end stage renal disease, diabetes, and depression	Yes or no	At least 2 diagnosis codes or 1 procedure code.
Gagne comorbidity index	<0, 1‐2, 3‐4, >4	Measure of comorbidity burden. Higher scores indicate more comorbidities [24].
Body mass index	<18.5, 18.5‐24.9, 25‐29.9, 30‐39.9, ≥40 kg/m^2^	Weight and height measured closest to and prior to first scan.
**Facility characteristics**		CDW
Facility complexity	1a (most complex), 1b, 1c, 2, 3 (low complexity)	Determined based on a model that considers clinical programs and patient risk levels, as well as research and teaching. The model is reviewed and updated with current data every 3 years.
**Use**		CDW; ascertained in the 2 years prior to baseline
Inpatient stays	0, 1+	
Telehealth encounters	Quartiles	Determined by stop codes, and includes visits that were designated as telephone, video, “tele,” or virtual

a CDW: corporate data warehouse.

b FY: fiscal year.

c RUCA: Rural Urban Commuting Area.

d VA: Department of Veterans Affairs.

e ADI: area deprivation index.

### Statistical Analyses

We calculated the percentage of patients who were noncompliant, with 95% CIs. To evaluate associations between characteristics and noncompliance, we estimated OR and corresponding 95% CIs using a logistic regression model that included all covariates. The Akaike information criterion (AIC) was used to assess contributions of each covariate and groups of covariates to model fit along with a likelihood ratio test for a model that excluded the covariate or group of covariates [25]. AIC helps to quantify how well a model fits the data it was generated from relative to other models fit on the same data. AIC penalizes models that use more parameters to reduce the potential for overfitting. Lower AIC scores are considered evidence of better model fit.

Missing data were recovered using multiple imputation by chained equations using all covariates and the outcome and results displayed are from pooling the 20 imputed datasets [26-28]. Generalized variance inflation factors [29] for each of the covariates were calculated to assess correlation between covariates, and the impact it may have had on regression results. A variance inflation factor of 4 or more was used as evidence of substantial collinearity [30]; there was no evidence of substantial collinearity.

We conducted several sensitivity analyses. First, because there is no empirical basis for our definition of noncompliance [31], and some prior studies have used a higher cutoff, we conducted analyses using different cut points of minimum days per week on average (2 and 3) and months in the past 12 (11, 9, and 6) for defining noncompliance. Second, we graphically explored the association between hospitalization and separately, amputation, on SmartMat use by examining use in the 6 months prior to, and the 6 months after a hospitalization or amputation (separately). In these analyses, we categorized days per week into 4 categories: no use, <2 days per week, 2 to <5 days per week, and 5‐7 days per week. We also graphed use over time in those with a hospitalization or amputation (separately) relative to those who had neither a hospitalization nor an amputation.

### Ethical Considerations

This program evaluation qualified as a nonresearch quality improvement activity conducted under the authority of VHA operations. It complies with the VHA definition of “non-research operations activities” outlined in section 5a of the 2019 *VHA Program Guide 1200.21: VHA Operations Activities That May Constitute Research*, meeting both specified conditions: (1) the evaluation was designed and implemented for internal VHA purposes and (2) not designed to produce information to expand the knowledge base of a scientific discipline.

## Results

The sample was predominantly male (n=1125, 98.94%); 21.1% (n=230) were Black and 75.7% (n=825) were White (Tables2  3). Just over half (n=595, 53.8%) of the patients were married. Nearly half (n=525, 46.2%) of the patients were aged between 70 and 79 years and 96.9% (n=1102) had diabetes. In the 2 years prior to baseline, 82.0% (n=932) had a diabetic foot ulcer, 40.4% (n=459) had osteomyelitis, 41.6% (n=473) had chronic kidney disease or end stage renal disease, 30.7% (n=349) had depression, and 53.3% (n=606) were hospitalized. Over a third (n=409, 36.0%) had a Gagne comorbidity of 4 or greater and 59.8% (n=660) had a BMI>30 kg/m^2^. About two-thirds of patients had poorly controlled diabetes based on a hemoglobin A_1c_ greater than or equal to 7.0, including 7% (n=80) with a hemoglobin A_1c_ greater than or equal to 10. Further, 31.2% (n=181) of patients were current smokers, though smoking status was missing for nearly half of the participants. Lastly, 74.9% (n=851) of the patients lived in urban areas and less than 20% (n=143, 12.6%) lived more than 30 minutes’ drive time from primary care or 60 minutes’ drive time from specialty care (n=182, 16%). Most of the patients came from the Midwest (n=361, 35.6%) or the Pacific region (n=335, 33.0%), while less than 5% (n=48) of the patients were from the Southeast. Multimedia Appendix 1 compares those included in analyses to those who were excluded because of insufficient follow-up. Briefly, the individuals with insufficient follow-up who were excluded from analyses had fewer ulcer risk factors, were predominantly from the Continental region, and were more likely to live in rural areas where they had longer drive times to primary and specialty care.

**Table 2.** Demographic, socioeconomic, and health characteristics of patients enrolled in remote foot temperature monitoring in the Department of Veterans Affairs between January 2019 and June 2021 (N=1137).^a^^,^^b^^,^^c^^,^^d^

Characteristic			Patients, n (%)
**Demographic or socioeconomic**			
	**Sex**		
		Female	12 (1)
		Male	1125 (98.9)
	**Race**		
		American Indian or Alaska Native	11 (1)
		Asian	1 (<1)
		Black	230 (21.1)
		More than one race	10 (1)
		Native Hawaiian or Other Pacific Islander	13 (1)
		White	825 (75.7)
	**Ethnicity**		
		Hispanic or Latino	77 (7)
		Not Hispanic or Latino	1038 (93.1)
	**Marital status**		
		Married	595 (53.8)
		Separated or divorced	317 (27.9)
		Single	136 (12.3)
		Widowed	58 (5)
	**Area deprivation index (national rank)^e^**		
		1‐24	232 (22.9)
		25‐49	281 (27.7)
		50‐74	256 (25.2)
		75+	245 (24.2)
		Unknown	123 (10.8)
**Health or comorbidities**			
	**Age (years)**		
		<50	17 (2)
		50‐59	123 (10.8)
		60‐69	353 (31.0)
		70‐79	525 (46.2)
		80+	119 (10.5)
	**Diabetes**		
		No	35 (3)
		Yes	1102 (96.9)
	**Nonhealing ulcer**		
		No	205 (18.0)
		Yes	932 (82.0)
	**Osteomyelitis**		
		No	678 (59.6)
		Yes	459 (40.4)
	**Chronic kidney disease or end stage kidney disease**		
		No	664 (58.4)
		Yes	473 (41.6)
	**Lower extremity amputation**		
		Neither	664 (58.4)
		Partial foot	213 (18.7)
		Major lower limb	260 (22.9)
	**Gagne comorbidity index**		
		≤0	319 (28.1)
		1‐2	219 (19.3)
		3‐4	190 (16.7)
		>4	409 (36.0)
	**Depression**		
		No	788 (69.3)
		Yes	349 (30.7)
	**Body mass index (kg/m** ^**2**^ **)**		
		<18.5	2 (<1)
		18.5‐24.9	136 (12.3)
		25‐29.9	305 (27.7)
		30‐39.9	547 (49.6)
		40+	113 (10.2)
	**Inpatient visits**		
		0	531 (46.7)
		1+	606 (53.3)
	**Telehealth encounters**		
		<6	141 (12.4)
		6‐12	234 (20.6)
		13‐27	361 (31.8)
		28+	401 (35.3)

a Noncompliance defined as mat use of <2 times per week for 4 or more of the 12 months of follow-up.

b Categories may not sum up to column total because of missing values.

c Percent calculated among those with nonmissing values.

d Unknown category presented if >5% of total sample.

e Lower area deprivation index indicates less deprivation.

**Table 3.** Behavioral, access to care, and practice patterns characteristics of patients enrolled in remote foot temperature monitoring in the Department of Veterans Affairs between January 2019 and June 2021 (N=1137).^a^^,^^b^^,^^c^^,^^d^

Characteristic			Patients, n (%)
**Behavioral**			
	**Hemoglobin A**_**1c**_		
		<5.7	72 (6)
		5.7‐6.9	298 (26.7)
		7‐7.9	318 (28.4)
		8‐9.9	315 (28.2)
		10+	80 (7)
		No diabetes	25 (2)
	**Smoking status**		
		Current smoker	181 (31.2)
		Former smoker	204 (35.1)
		Never smoker	196 (33.7)
		Unknown	556 (48.9)
	**Substance use disorder**		
		No	905 (79.6)
		Yes	232 (20.4)
**Access to care**			
	**Rurality**		
		Rural or highly rural	286 (25.2)
		Urban	851 (74.8)
	**Drive time (primary care)**		
		<30 min	991 (87.4)
		30+ min	143 (12.6)
	**Drive time (specialty care)**		
		<60 min	952 (84.0)
		60+ min	182 (16.0)
**Practice patterns**			
	**Department of Veterans Affairs district or region**		
		Continental	131 (12.9)
		Midwest	361 (35.6)
		North Atlantic	139 (13.7)
		Pacific	335 (33.0)
		Southeast	48 (5)
		Unknown	123 (10.8)
	**Facility complexity**		
		1a—high complexity	433 (42.7)
		1b—high complexity	315 (31.1)
		1c—high complexity	135 (13.3)
		2—medium complexity	71 (7)
		3—low complexity	60 (6)
		Unknown	123 (10.8)

a Noncompliance defined as mat use of <2 times per week for 4 or more of the 12 months of follow-up.

b Categories may not sum up to column total because of missing values.

c Percent calculated among those with nonmissing values.

d Unknown category presented if >5% of total sample.

**Figure 1.**
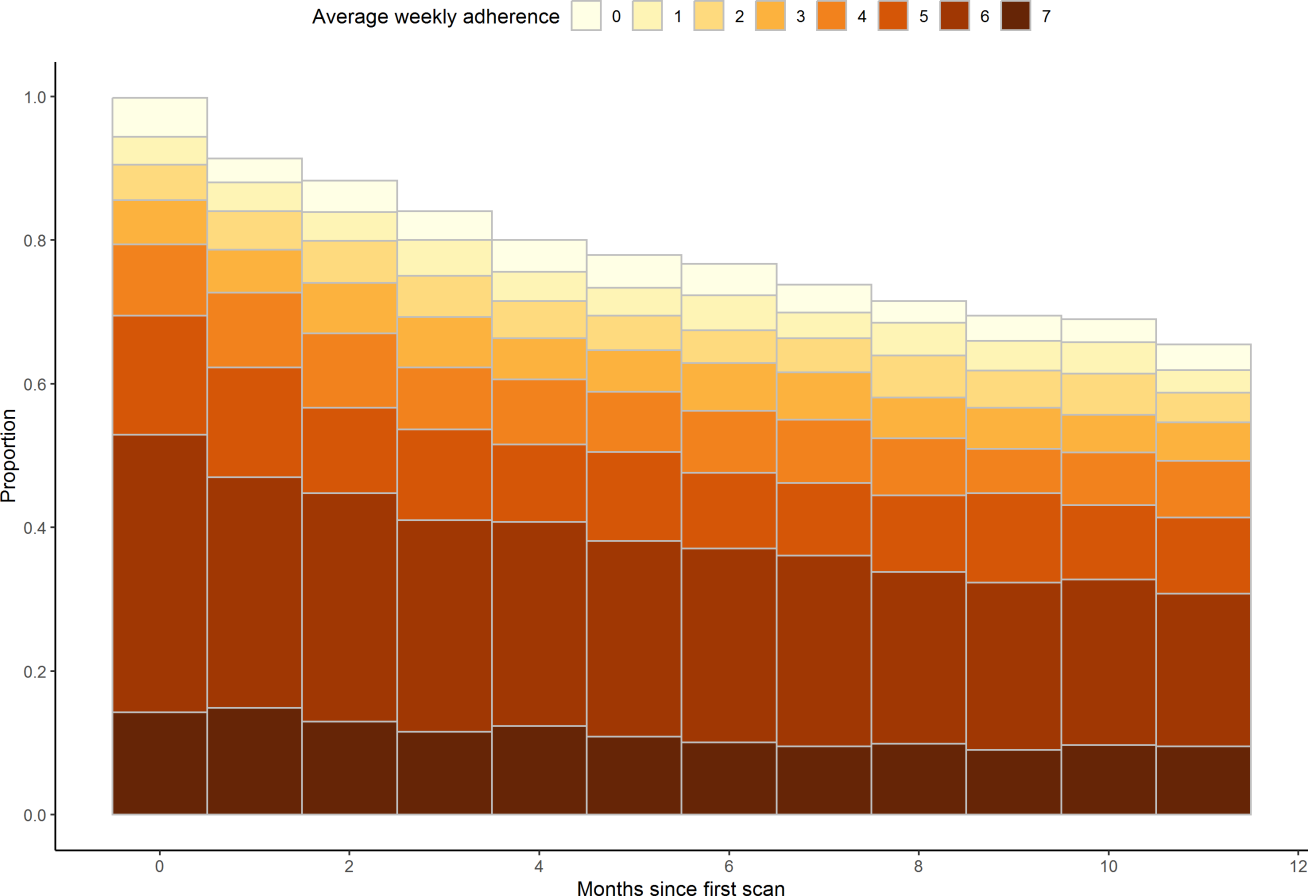
Average days per week remote temperature monitoring was used. Note that the group marked “0” is >0 but less than 1. Individuals who did not use the mat at all in the month are represented by the absence of any bars.

Overall, 37.6% (428/1137) of patients were classified as noncompliant (Tables4^6^ and Figure 1). Mat use declined over time; by month 12, over 30% of patients never used the mat in the prior month. In descriptive analyses, the factors associated with higher noncompliance (5 percentage points or more above the mean) included race other than Black or White (43% noncompliance), Hispanic or Latino ethnicity (48%), widowed (47%), ADI <24 (47%), age <60 (47% for those aged <50 years and 44% for those aged 50‐59 years), osteomyelitis (45%), major lower limb amputation (44%), Gagne comorbidity index >4 (48%), depression (44%), BMI <25 kg/m^2^ (49%), inpatient visit in the 2 years before baseline (43%), more than 28 telehealth encounters (43%), hemoglobin A_1c_ ≥10 (60%), current smoking (54%), substance use disorder (53%), living in the Pacific region (44%), and receiving care at a high-complexity facility (44%). The factors associated with *lower* noncompliance (5 percentage points or more below the mean) included being a female (33% noncompliance) and living in an area with an ADI between 50 and 74 (31%); *not* having a recent history of ulceration (32%), osteomyelitis (33%), or hospitalization (31%); and having a lower comorbidity index score (≤0: 29% noncompliance; 1‐2: 30% noncompliance); BMI ≥30 kg/m^2^ (30‐39.9 kg/m^2^: 33% noncompliance; ≥40 kg/m^2^: 30% noncompliance) fewer than 12 telehealth encounters (<6: 28% noncompliance, 6‐12: 31% noncompliance); and hemoglobin A_1c_ between 7 and 7.9 (32% noncompliance). Lastly, noncompliance was lower among those from the Midwest (32%) and at low-complexity facilities (22%). In the multivariable model, compared to an ADI <25, intermediate ADI was inversely associated with noncompliance (ADI 50‐74: OR 0.56, 95% CI 0.35-0.89); associations for the other categories were not statistically significantly different from the lowest ADI category. Recent history of osteomyelitis (OR 1.44, 95% CI 1.06‐1.97), Gagne comorbidity index score ≥4 (vs ≤0: OR 1.81, 95% CI 1.15-2.83), telehealth encounters (13‐27 vs <6: OR 1.65, 95% CI 1.01-2.70; 28+ vs <6: OR 1.70, 95% CI 1.02-2.84), hemoglobin A_1c_ ≥10 (vs <5.7: OR 2.67, 95% CI 1.27-5.58), and current smoking (vs never smoking: OR 2.06, 95% CI 1.32-3.20) were statistically significantly and positively associated with noncompliance. Using AIC to help inform the contribution of different variables to model fit, behavioral factors (hemoglobin A_1c_ and smoking), and to a lesser extent, health conditions or comorbidities (eg, osteomyelitis and Gagne comorbidity index) most contributed to model fit (Table 7). The results were not meaningfully different in the sensitivity analyses that used different cut points for minimum number of days per week and months in the past 12 to define noncompliance (data not presented).

**Table 4.** Estimated associations^a^ of demographic or socioeconomic characteristics with remote foot temperature monitoring noncompliance^b^ within conceptual groups using multiply imputed data (N=1137).

Demographic or socioeconomic characteristics		Noncompliant (%)	95% CI	Adjusted odds ratio	95% CI
**Sex**					
	Female	33	14‐61	0.69	0.18‐2.65
	Male	38	35‐41	1.00	Reference
**Race**					
	Black	42	36‐49	1.34	0.92‐1.95
	Race other than Black or white	43	28‐59	1.23	0.57‐2.65
	White	36	33‐39	1.00	Reference
**Ethnicity**					
	Hispanic or Latino	48	37‐59	1.46	0.86‐2.47
	Not Hispanic or Latino	37	34‐40	1.00	Reference
**Marital status**					
	Married	35	32‐39	1.00	Reference
	Separated or divorced	40	35‐46	1.10	0.79‐1.52
	Single	40	32‐48	0.94	0.60‐1.46
	Widowed	47	34‐59	1.28	0.69‐2.38
**Area deprivation index national rank** ^**c**^					
	1‐24	47	41‐53	1.00	Reference
	25‐49	41	35‐46	0.77	0.51‐1.16
	50‐74	31	26‐37	0.56	0.35‐0.89
	75+	37	31‐43	0.65	0.39‐1.08

a A single multivariable model was used to estimate adjusted odds ratios; each factor was adjusted for all of the other factors in the model.

b Noncompliance defined by average weekly mat use of <2 days per week for at least 4 of the 12 months of follow-up.

c Lower area deprivation index indicates less deprivation.

**Table 5.** Estimated associations^a^ of health characteristics with remote foot temperature monitoring noncompliance^b^ within conceptual groups using multiply imputed data (N=1137).

Health or comorbidities characteristics		Noncompliant (%)	95% CI	Adjusted odds ratio	95% CI
**Age (years)**					
	<50	47	26‐69	1.49	0.49‐4.48
	50‐59	44	35‐53	1.19	0.75‐1.89
	60‐69	38	33‐43	0.86	0.62‐1.19
	70‐79	35	31‐39	1.00	Reference
	80+	40	32‐49	1.48	0.92‐2.38
**Nonhealing ulcer**					
	No	32	26‐38	1.00	Reference
	Yes	39	36‐42	1.02	0.68‐1.52
**Osteomyelitis**					
	No	33	29‐36	1.00	Reference
	Yes	45	40‐49	1.44	1.06‐1.97
**Chronic kidney disease or end stage renal disease**					
	No	35	34‐42	1.00	Reference
	Yes	41	36‐45	0.89	0.65‐1.23
**Lower extremity amputation**					
	Neither	35	31‐38	1.00	Reference
	Partial foot	38	32‐45	1.01	0.70‐1.46
	Major lower limb	44	38‐50	1.11	0.77‐1.60
**Gagne index**					
	≤0	29	24‐34	1.00	Reference
	1‐2	30	24‐36	0.78	0.50‐1.21
	3‐4	40	33‐47	1.37	0.86‐2.18
	>4	48	43‐53	1.81	1.15‐2.83
**Depression**					
	No	35	32‐38	1.00	Reference
	Yes	44	38‐49	1.19	0.87‐1.62
**Body mass index (kg/m** ^**2**^ **)**					
	<25	49	41‐58	1.00	Reference
	25‐29.9	42	37‐48	0.89	0.57‐1.40
	30‐39.9	33	29‐37	0.70	0.45‐1.08
	40+	30	22‐39	0.73	0.40‐1.33
**Inpatient visits**					
	0	31	27‐35	1.00	Reference
	1+	43	40‐47	0.89	0.62‐1.28
**Telehealth encounters**					
	<6	28	21‐36	1.00	Reference
	6‐12	31	26‐37	1.19	0.71‐1.99
	13‐27	40	35‐45	1.65	1.01‐2.70
	28+	43	38‐48	1.70	1.02‐2.84

a A single multivariable model was used to estimate adjusted odds ratios; each factor was adjusted for all of the other factors in the model.

b Noncompliance defined by average weekly mat use of <2 days per week for at least 4 of the 12 months of follow-up.

**Table 6.** Estimated associations^a^ of behavioral, access to care, and practice pattern characteristics with remote foot temperature monitoring noncompliance^b^ within conceptual groups using multiply imputed data (N=1137).

Characteristic			Noncompliant (%)	95% CI	Adjusted odds ratio	95% CI
**Behavioral**						
	**Hemoglobin A**_**1c**_					
		<5.7	42	31‐53	1.00	Reference
		5.7‐6.9	35	30‐40	0.90	0.50‐1.62
		7‐7.9	32	27‐38	0.89	0.49‐1.61
		8‐9.9	39	34‐45	1.14	0.63‐2.05
		10+	60	49‐70	2.67	1.27‐5.58
		No diabetes	34	21‐51	0.96	0.37‐2.44
	**Smoking status**					
		Current smoker	54	47‐61	2.06	1.32‐3.20
		Former smoker	32	26‐39	0.96	0.62‐1.47
		Never smoker	35	29‐42	1.00	Reference
	**Substance use disorder**					
		No	34	31‐37	1.00	Reference
		Yes	53	47-60	1.33	0.90‐1.97
**Access to care**						
	**Rurality**					
		Highly rural or rural	36	31‐42	1.10	0.73‐1.64
		Urban	38	35‐41	1.00	Reference
	**Drive time primary care**					
		<30 min	38	35‐41	1.00	Reference
		30+ min	34	27‐42	0.90	0.55‐1.49
	**Drive time specialty care**					
		<60 min	37	34‐41	1.00	Reference
		60+ min	38	32‐46	1.04	0.69‐1.58
**Practice patterns**						
	**Department of Veterans Affairs district or region**					
		Continental	38	30‐47	1.00	Reference
		Midwest	32	27‐37	0.90	0.54‐1.49
		North Atlantic	42	35‐51	1.46	0.82‐2.58
		Pacific	44	39‐50	1.22	0.71‐2.10
		Southeast	40	27‐54	1.94	0.84‐4.49
	**Facility complexity**					
		1a—high complexity	44	39‐49	1.00	Reference
		1b—high complexity	37	32‐43	0.89	0.56‐1.40
		1c—high complexity	34	26‐42	0.88	0.50‐1.56
		2—medium complexity	34	24‐45	0.80	0.41‐1.55
		3—low complexity	22	13‐34	0.50	0.23‐1.06

a A single multivariable model was used to estimate adjusted odds ratios; each factor was adjusted for all of the other factors in the model.

b Noncompliance defined by average weekly mat use of <2 days per week for at least 4 of the 12 months of follow-up.

**Table 7.** Summary of AIC^a^ for conceptual groups and variables for association with noncompliance^b^.

Conceptual group	AIC	AIC difference versus saturated model	Likelihood ratio test *P* value^c^
**Saturated model**	1444	Reference	—^d^
** Demographics**	1437	−7	.30
Sex	1442	−2	.57
Race	1443	−1	.30
Hispanic or Latinx ethnicity	1444	0	.15
Marital status	1439	−5	.78
Area deprivation index	1445	+1	.11
**Health conditions or comorbidities**	1461	+17	<.001
Age	1442	−2	.23
Ulcer	1442	−2	.93
Osteomyelitis	1447	+3	.03
Chronic kidney disease or end stage renal Disease	1443	−1	.48
Lower extremity amputation	1440	−4	.84
Gagne comorbidity index	1454	+10	.002
Depression	1443	−1	.29
Body mass index	1442	−2	.28
Inpatient visits	1442	−2	.55
Telehealth encounters	1445	+1	.10
**Behavioral**	1473	+29	<.001
Hemoglobin A_1c_	1452	+8	.005
Smoking	1460	+16	.007
Substance use disorder	1444	0	.11
**Access to care**	1438	−6	.95
Rurality	1442	−2	.65
30+ min to primary care	1442	−2	.67
60+ min to specialty care	1442	−2	.86
**Practice patterns**	1443	−1	.15
District	1445	+1	.15
Facility complexity	1441	−3	.46

a AIC: Akaike information criterion.

b Larger Akaike information criterion difference versus saturated model indicate more substantial contribution to the model fit.

c *P* values were calculated using a modification for multiply imputed data [25].

d Not applicable.

When considering changes in use around the time of a hospitalization or amputation, we found that the fraction of people with 5‐7 days per week of SmartMat use decreased, while the proportion of those with no SmartMat use increased in the month of the hospitalization or amputation (MultimediaAppendices 2  3). The proportion with *no* SmartMat use increased slightly in the 6 months after the hospitalization or amputation (MultimediaAppendices 4  5). Specifically, there was a larger increase in the proportion of patients with no use (and decrease in the proportion of patients with 5‐7 d of use) in the month concurrent with a hospitalization or amputation as well as in the 6 months after, compared to the reference group of patients with no hospitalization or amputation during follow-up.

## Discussion

### Principal Findings

Even though the requirements of monitoring are minimal (20 s per day to obtain a scan), nearly 4 in 10 patients did not use the SmartMat at least 2 days per week for at least 75% of the months under observation. Based on AIC differences, the strongest factors associated with noncompliance were behavioral factors (poor glucose control [as measured by hemoglobin A_1c_] and smoking), and to a lesser extent, factors such as a recent history of osteomyelitis and an elevated Gagne comorbidity index, indicating a high comorbidity burden. Patients who are unable to manage their blood glucose levels or quit smoking, as well as those with osteomyelitis and numerous chronic comorbidities may also have challenges regularly using a home temperature monitoring device. Noncompliance was also higher among those who had a hospitalization or amputation. Although some results were different in descriptive analyses (eg, BMI and ADI) or statistically significant (eg, ADI) in our multivariable model, the absence of large AIC differences indicated that they did not contribute importantly to model fit and therefore may not be important factors for compliance. Additional research that replicates these findings and that can help us understand reasons for noncompliance in patients would be helpful to inform future interventions.

We are only aware of a single study [21] that evaluated compliance with a SmartMat for foot temperature monitoring, and that study found lower noncompliance using a more stringent definition. Unlike our study, the Frykberg et al [21] study excluded days during which a participant had a contraindication to using the mat (eg, for an open plantar wound) and considered those who did not use the mat for >28 consecutive days as lost to follow-up (18.6% of the sample) [21]. As our sensitivity analyses indicated a reduction in use following a hospitalization or amputation, differences in use between the prior study and ours may be partly because we did not exclude any days. In any case, noncompliance in this study is similar to prior studies of foot temperature monitoring involving handheld thermometers. For example, in a trial of 151 people randomized to daily foot temperature monitoring using a handheld thermometer, 62% of participants measured foot temperatures at least 70% of days (equivalent to 38% noncompliance) [14]. Likewise, a study of daily temperature monitoring in Peru observed 60% compliance when treating those who did not return logbooks as nonadherent (vs adherence of 87% when leaving them out of the analysis, equivalent to 40% and 13% noncompliance) [32]. Estimates for foot temperature monitoring are also similar to general adherence to a variety of self-management activities in people with diabetes observed in meta-analyses [33]. In summary, even though RTM is a relatively low burden intervention, noncompliance rates are strikingly similar despite different definitions of adherence and different activity or intervention burdens, suggesting that factors other than time burden likely impact compliance.

The Unified Theory of Acceptance and Use of Technology 2 (UTAUT2) [34] is a helpful framework for understanding why individuals accept and use technology and may help to understand factors impacting compliance with RTM. UTAUT2 outlines how 7 constructs influence consumers’ intention to use a technology: (1) performance expectancy (does it help?), which is analogous to relative advantage in the diffusion of innovation literature [35] and perceived usefulness in the technology acceptance model [36], (2) effort expectancy (how easy is it to use?), (3) facilitating conditions (are there resources available to support use?), (4) social influence (do those close to the individual support their use of the technology?), (5) hedonic motivation (is it fun?), (6) price value (is it worth it financially?), and (7) habit (does it become a habit?).

Because we lack data from patients’ perspectives, it is unclear whether patients perceived a benefit, especially those who did not have any hot spots, or had many hot spots. Unlike a scale or a blood pressure monitoring device, which provides direct feedback with each use, and a sense of accomplishment for those who are losing weight or lowering their blood pressure, patients were not routinely provided direct feedback. They were called if they did not use the mat, or if temperature asymmetries were detected. If perceived benefit is low, use may decrease over time. Even though the apparent time burden is low, there may be steps (such as removing socks and shoes) that may be challenging and prevent people from using the mat more regularly. Given that an annual SmartMat subscription includes access to company personnel to answer questions, the third construct from the UTAUT2, facilitating conditions, may be high. As social influence is known to be important in diabetes management [37], social influence may be an important factor for this technology. Unfortunately, we had no direct measures of social influence, and marital status (a poor proxy) was not associated with compliance, which is understandable because marital status does not provide a direct measure of whether someone has a positive social influence. RTM is intended for disease management and was not designed to be fun, so hedonic motivation may be low. Gamification (eg, points and badges for streaks or other goals) could make it more fun and might improve compliance [38]. Price value is likely low since VA provides the SmartMat free of charge to patients. Future studies that collect self-report information from patients, including patient interviews, could help elucidate the extent to which factors in the model facilitate use.

### Strengths and Limitations

A major strength of our study was its large size and ability to examine associations between various characteristics and SmartMat noncompliance. A potential limitation is that we did not have data on each day’s use, but instead an average number of days per week per month of follow-up. Though less precise, this level of detail is informative to understand trends. Third, there are no standard definitions of adherence or noncompliance, and our definition was different from the 1 used in a prior study [21], though it was based on expert opinion. Fourth, we lacked information on factors not readily available in the medical record such as patient preferences, perceived benefits, beliefs, attitudes, social support, and environmental factors that may have impacted use of the mats. This information would be useful to collect in future studies. Finally, because our sample included VA patients, individuals were primarily older White males, so results may not generalize to more diverse samples. Future research, particularly randomized trials testing different approaches to increase compliance (eg, gamification, incentives, patient and caregiver education, motivational interviewing, and reminders or alerts) in different patient populations, will be valuable to informing how to make technologies such as these more impactful.

### Conclusion

Our study found that a large fraction of patients did not use the SmartMat as directed, and thus they would be unlikely to benefit from it. Testing approaches to proactively provide additional support for self-monitoring to patients with poor glucose control, current smoking, or high comorbidity burden (factors associated with high noncompliance) is an important area of future research. Future research should also seek to understand patients’ perspectives on their experience with SmartMats and why they may have routinely used, rarely used, or stopped using the mat. Reducing the risk of ulcer recurrence and amputation could have enormous benefits for individual patients and lower health care costs. Thus, ensuring that patients effectively employ tools to reduce the risk of ulcer recurrence is paramount.

## Supplementary material

10.2196/53083 Multimedia Appendix 1 Patient characteristics by study exclusion (N=1641).

10.2196/53083 Multimedia Appendix 2 Average days per week remote temperature monitoring was used among patients with a hospitalization during follow-up (n=477). Note that month 0 represents the month of their hospitalization and negative numbers on the x axis are months prior to their hospitalization and positive numbers are months after.

10.2196/53083 Multimedia Appendix 3 Average days per week remote temperature monitoring was used among patients with an amputation during follow-up (n=97). Note that month 0 represents the month of their amputation and negative numbers on the x axis are months prior to their amputation and positive numbers are months after.

10.2196/53083 Multimedia Appendix 4 Average days per week remote temperature monitoring was used among patients with a hospitalization during follow-up (n=477) compared to a reference group includes those who did not have a hospitalization or amputation during follow-up.

10.2196/53083 Multimedia Appendix 5 Average days per week remote temperature monitoring was used among patients with an amputation during follow-up (n=97) compared to a reference group that includes those who did not have a hospitalization or amputation during follow-up.

## Abbreviations

ADI: area deprivation index
AIC: Akaike information criterion
CDW: corporate data warehouse
FY: fiscal year
OR: odds ratio
RTM: remote foot temperature monitoring
RUCA: Rural Urban Commuting Area
UTAUT2: Unified Theory of Acceptance and Use of Technology 2
VA: Department of Veterans Affairs
VHA: Veterans Health Administration
