# Supplementary material for: Remote Foot Temperature Monitoring Among Veterans: Large Observational Study of Noncompliance and Its Correlates
Source: JMIR Diabetes. 2024 Nov 5;9:e53083. doi: 10.2196/53083 (PMC11555900; doi:10.2196/53083)
Supplement: Multimedia Appendix 1 [file diabetes-v9-e53083-s001.docx]

**Table S1.** Patient characteristics by study exclusion (N=1641).

| **Characteristic** | **Excluded because of insufficient follow-up time (n=504)** | | **Retained (n=1137)** | |
| --- | --- | --- | --- | --- |
|  | **n** | **%** | **n** | **%** |
| **Demographic/SES** |  |  |  |  |
| **Sex** |  |  |  |  |
| Female | 16 | 3.2% | 12 | 1.1% |
| Male | 488 | 96.8% | 1125 | 98.9% |
| **Race** |  |  |  |  |
| Black or African American | 93 | 18.5% | 230 | 20.2% |
| Other | 16 | 3.2% | 35 | 3.1% |
| White | 378 | 75.0% | 825 | 72.6% |
| *Unknown* | *17* | *3.4%* | *47* | *4.1%* |
| **Ethnicity** |  |  |  |  |
| Hispanic or Latino | 18 | 3.6% | 77 | 6.8% |
| Not Hispanic or Latino | 476 | 94.4% | 1038 | 91.3% |
| *Unknown* | *10* | *2.0%* | *22* | *1.9%* |
| **Marital status** |  |  |  |  |
| Married | 265 | 52.6% | 595 | 52.3% |
| Separated/divorced | 145 | 28.8% | 317 | 27.9% |
| Single | 52 | 10.3% | 136 | 12.0% |
| Widowed | 24 | 4.8% | 58 | 5.1% |
| *Unknown* | *18* | *3.6%* | *31* | *2.7%* |
| **Area deprivation index (national rank)** |  |  |  |  |
| 1-24 | 65 | 12.9% | 232 | 20.4% |
| 25-49 | 126 | 25.0% | 281 | 24.7% |
| 50-74 | 115 | 22.8% | 256 | 22.5% |
| 75+ | 104 | 20.6% | 245 | 21.5% |
| *Unknown* | *94* | *18.7%* | *123* | *10.8%* |
| **Health/comorbidities** |  |  |  |  |
| **Age** |  |  |  |  |
| <50 | 10 | 2.0% | 17 | 1.5% |
| 50-59 | 67 | 13.3% | 123 | 10.8% |
| 60-69 | 163 | 32.3% | 353 | 31.0% |
| 70-79 | 221 | 43.8% | 525 | 46.2% |
| 80+ | 43 | 8.5% | 119 | 10.5% |
| **Diabetes** |  |  |  |  |
| No | 39 | 7.7% | 35 | 3.1% |
| Yes | 465 | 92.3% | 1102 | 96.9% |
| **Non-healing ulcer** |  |  |  |  |
| No | 146 | 29.0% | 205 | 18.0% |
| Yes | 358 | 71.0% | 932 | 82.0% |
| **Osteomyelitis** |  |  |  |  |
| No | 341 | 67.7% | 678 | 59.6% |
| Yes | 163 | 32.3% | 459 | 40.4% |
| **Chronic kidney disease/ end stage kidney disease** |  |  |  |  |
| No | 309 | 61.3% | 664 | 58.4% |
| Yes | 195 | 38.7% | 473 | 41.6% |
|  |  |  |  |  |
| **Lower extremity amputation** |  |  |  |  |
| Neither | 352 | 69.8% | 664 | 58.4% |
| Partial foot | 71 | 14.1% | 213 | 18.7% |
| Major lower limb | 81 | 16.1% | 260 | 22.9% |
| **Gagne comorbidity index** |  |  |  |  |
| <=0 | 165 | 32.7% | 319 | 28.1% |
| 1-2 | 103 | 20.4% | 219 | 19.3% |
| 3-4 | 81 | 16.1% | 190 | 16.7% |
| >4 | 155 | 30.8% | 409 | 36.0% |
| **Depression** |  |  |  |  |
| No | 340 | 67.5% | 788 | 69.3% |
| Yes | 164 | 32.5% | 349 | 30.7% |
| **Body mass index (kg/m^2^)** |  |  |  |  |
| <18.5 | 1 | 0.2% | 2 | 0.2% |
| 18.5-24.9 | 51 | 10.1% | 136 | 12.0% |
| 25.0-29.9 | 114 | 22.6% | 305 | 26.8% |
| 30.0-39.9 | 234 | 46.4% | 547 | 48.1% |
| 40.0+ | 56 | 11.1% | 113 | 9.9% |
| *Unknown* | *48* | *9.5%* | *34* | *3.0%* |
| **Inpatient visits** |  |  |  |  |
| 0 | 256 | 50.8% | 531 | 46.7% |
| 1+ | 248 | 49.2% | 606 | 53.3% |
| **Telehealth encounters** |  |  |  |  |
| <6 | 33 | 6.5% | 141 | 12.4% |
| 6-12 | 76 | 15.1% | 234 | 20.6% |
| 13-27 | 154 | 30.6% | 361 | 31.8% |
| 28+ | 241 | 47.8% | 401 | 35.3% |
| **Behavioral** |  |  |  |  |
| **Hemoglobin A1C** |  |  |  |  |
| <5.7 | 16 | 3.2% | 72 | 6.3% |
| 5.7-6.9 | 140 | 27.8% | 298 | 26.2% |
| 7.0-7.9 | 155 | 30.8% | 318 | 28.0% |
| 8.0-9.9 | 118 | 23.4% | 315 | 27.7% |
| 10.0+ | 32 | 6.3% | 80 | 7.0% |
| No diabetes | 39 | 7.7% | 35 | 3.1% |
| Unknown | 4 | 0.8% | 19 | 1.7% |
| **Smoking status** |  |  |  |  |
| Current smoker | 95 | 18.8% | 181 | 15.9% |
| Former smoker | 36 | 7.1% | 204 | 17.9% |
| Never smoker | 33 | 6.5% | 196 | 17.2% |
| *Unknown* | *340* | *67.5%* | *556* | *48.9%* |
| **Substance use disorder** |  |  |  |  |
| No | 424 | 84.1% | 905 | 79.6% |
| Yes | 80 | 15.9% | 232 | 20.4% |
| **Practice patterns** |  |  |  |  |
| **VA District** |  |  |  |  |
| Continental | 207 | 41.1% | 131 | 11.5% |
| Midwest | 61 | 12.1% | 361 | 31.8% |
| North Atlantic | 29 | 5.8% | 139 | 12.2% |
| Pacific | 96 | 19.0% | 335 | 29.5% |
| Southeast | 13 | 2.6% | 48 | 4.2% |
| Unknown | 98 | 19.4% | 123 | 10.8% |
| **Facility complexity** |  |  |  |  |
| 1a-High Complexity | 148 | 29.4% | 433 | 38.1% |
| 1b-High Complexity | 154 | 30.6% | 315 | 27.7% |
| 1c-High Complexity | 24 | 4.8% | 135 | 11.9% |
| 2-Medium Complexity | 26 | 5.2% | 71 | 6.2% |
| 3-Low Complexity | 54 | 10.7% | 60 | 5.3% |
| *Unknown* | *98* | *19.4%* | *123* | *10.8%* |
| **Access to care** |  |  |  |  |
| **Rurality** |  |  |  |  |
| Rural/Highly rural | 180 | 35.7% | 286 | 25.2% |
| Urban | 324 | 64.3% | 851 | 74.8% |
| **Drive time (primary care)** |  |  |  |  |
| <30 minutes | 373 | 74.0% | 991 | 87.2% |
| 30+ minutes | 126 | 25.0% | 143 | 12.6% |
| *Unknown* | *5* | *1.0%* | *3* | *0.3%* |
| **Drive time (specialty care)** |  |  |  |  |
| <60 minutes | 374 | 74.2% | 952 | 83.7% |
| 60+ minutes | 125 | 24.8% | 182 | 16.0% |
| *Unknown* | *5* | *1.0%* | *3* | *0.3%* |
