# Supplementary figures and images for: Remote Foot Temperature Monitoring Among Veterans: Large Observational Study of Noncompliance and Its Correlates
Source: JMIR Diabetes. 2024 Nov 5;9:e53083. doi: 10.2196/53083 (PMC11555900; doi:10.2196/53083)

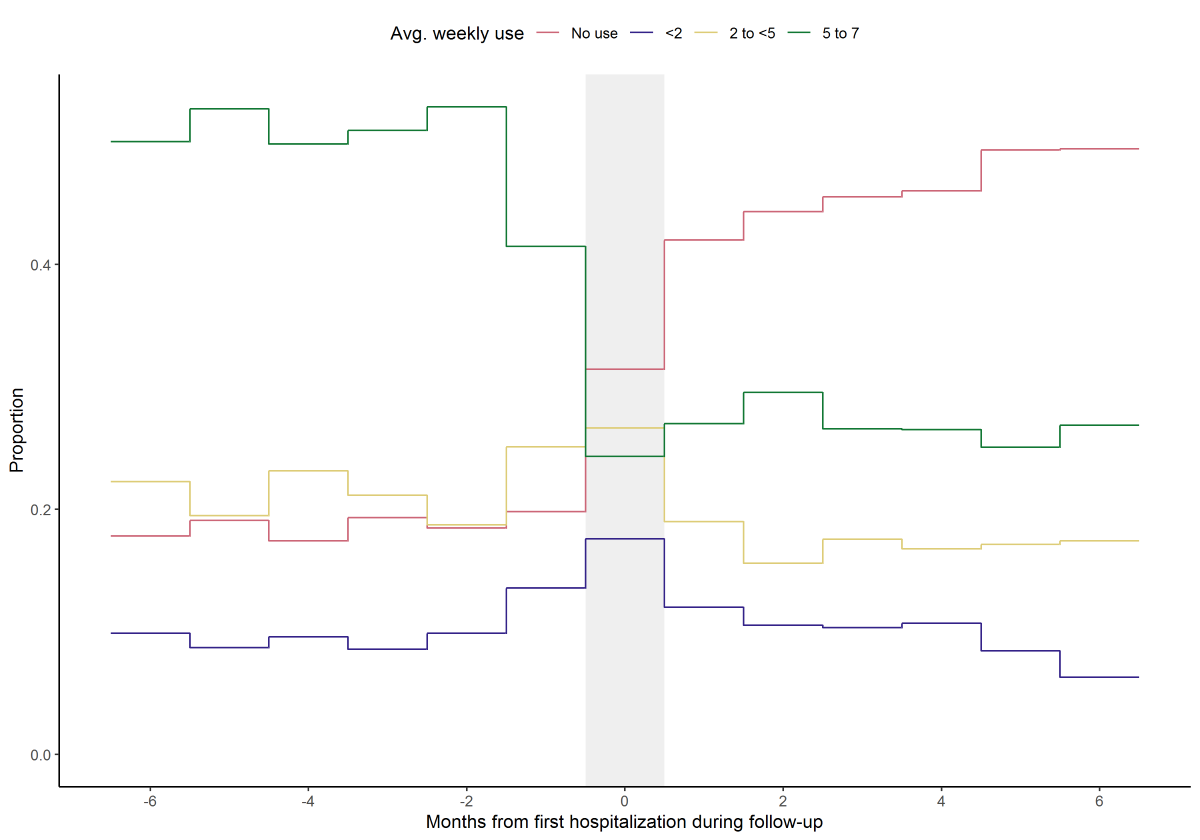

Supplement: Multimedia Appendix 2 [file diabetes-v9-e53083-s002.png]

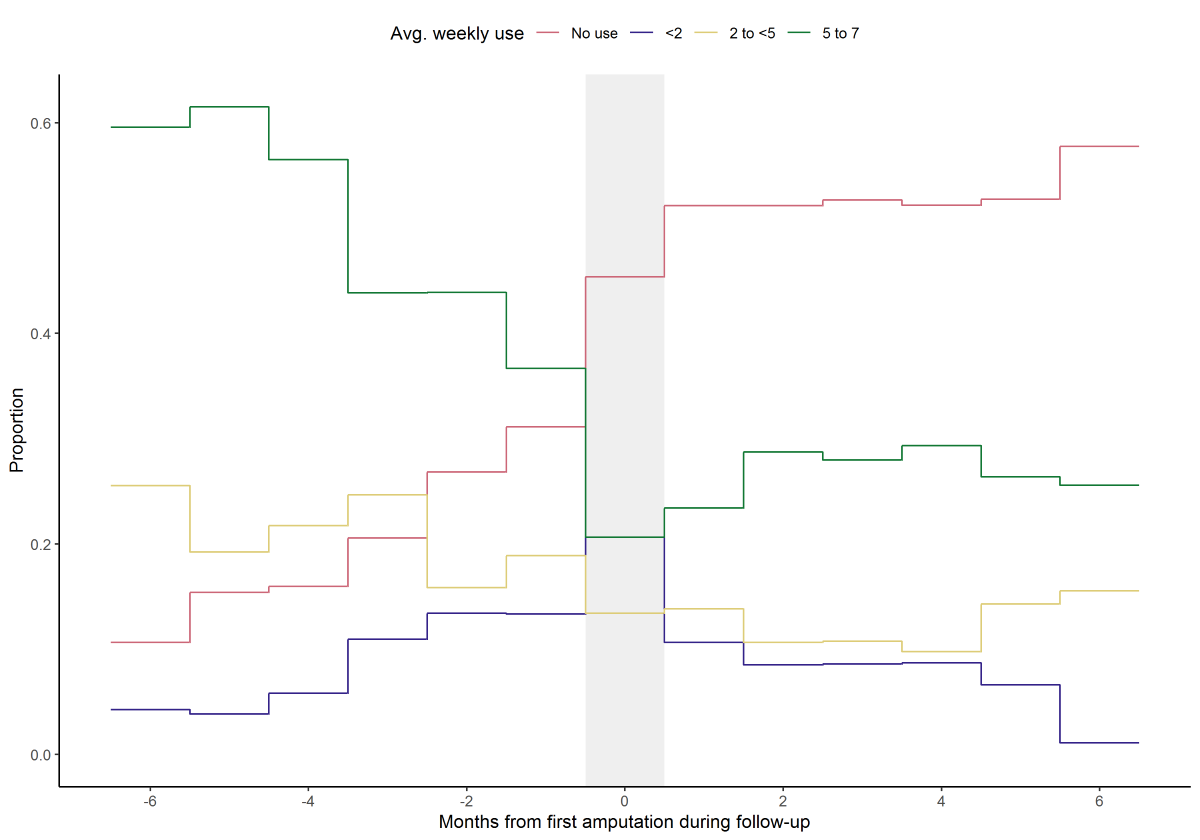

Supplement: Multimedia Appendix 3 [file diabetes-v9-e53083-s003.png]

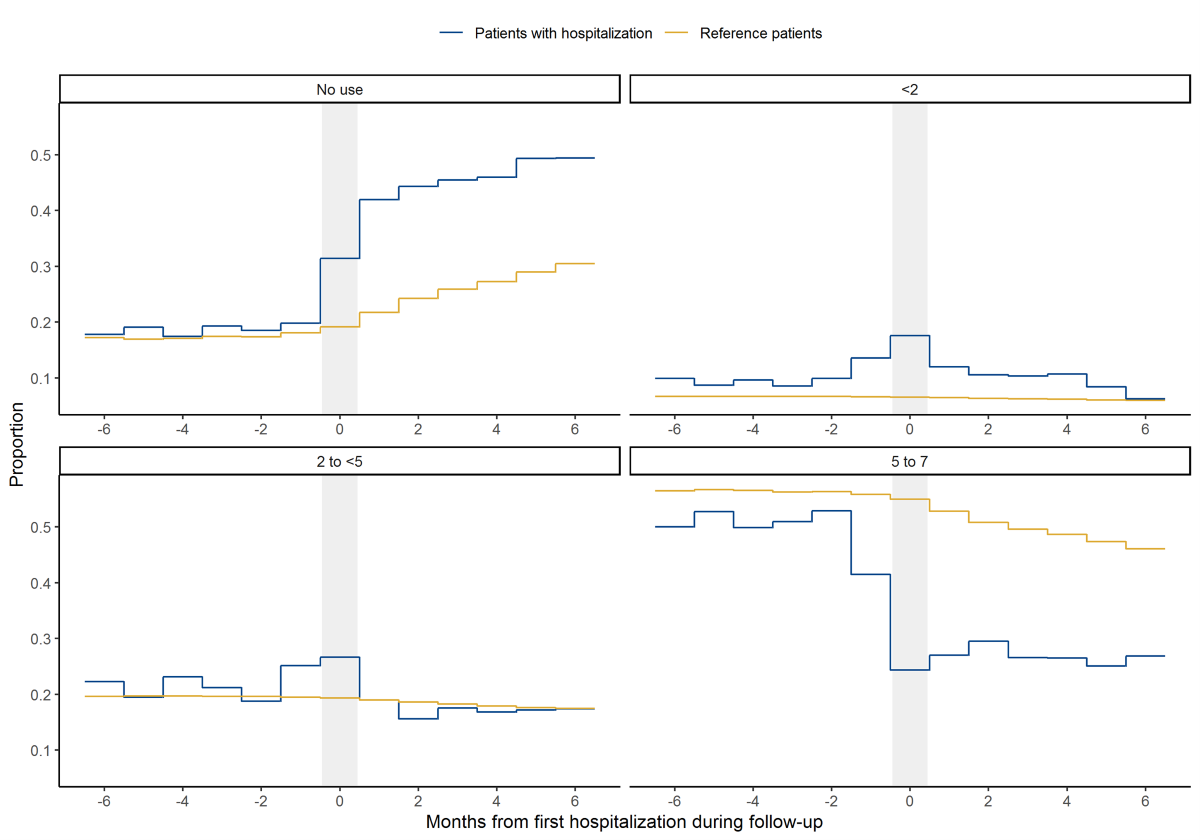

Supplement: Multimedia Appendix 4 [file diabetes-v9-e53083-s004.png]

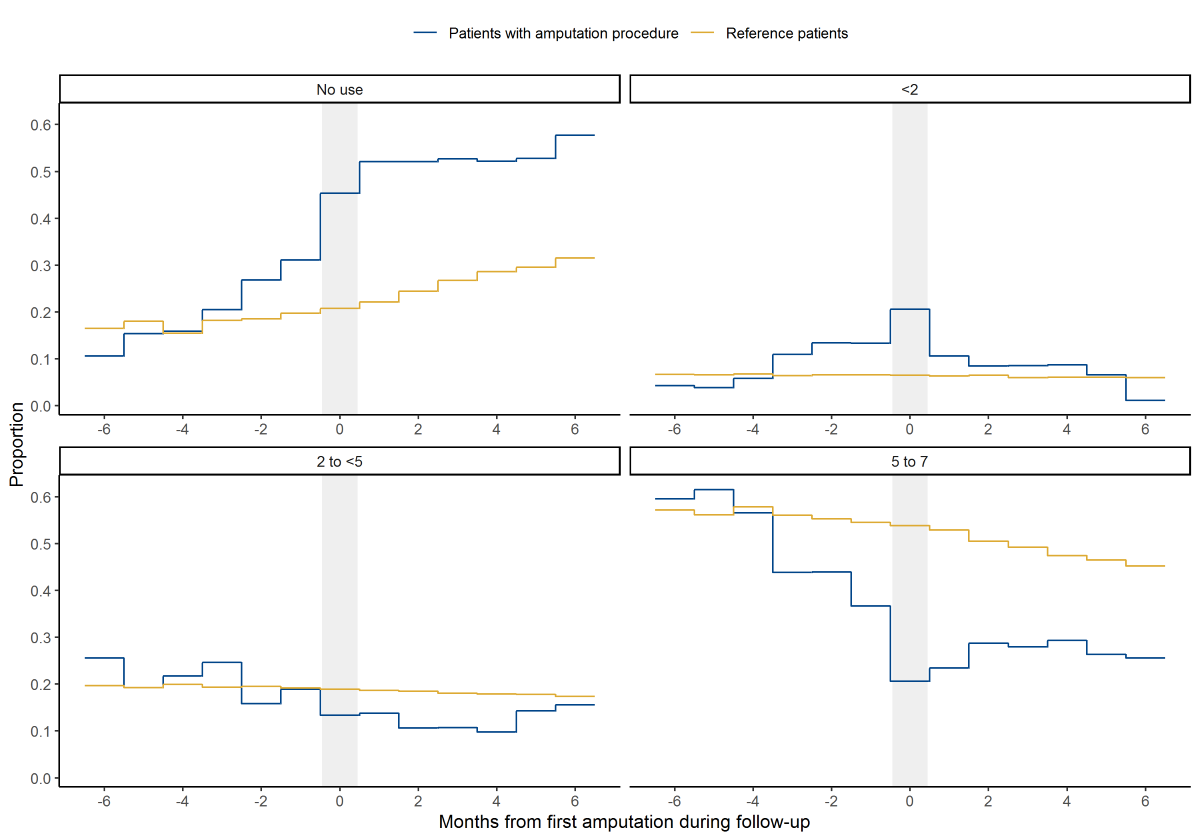

Supplement: Multimedia Appendix 5 [file diabetes-v9-e53083-s005.png]
